# Comparison of two automated ApoE4 proteotype immunoassays

**DOI:** 10.1002/alz70856_100528

**Published:** 2025-12-25

**Authors:** Daniel Figdore, Susan Ashrafzadeh‐Kian, Joshua A Bornhorst, Alicia Algeciras‐Schimnich

**Affiliations:** ^1^ Mayo Clinic, Rochester, MN, USA

## Abstract

**Background:**

The polymorphic *APOE* gene is a susceptibility gene for Alzheimer's disease (AD). The ε4 allele is associated with increased risk for AD, particularly late‐onset disease, in a dose‐dependent manner. Additionally, homozygous ε4/ε4 individuals have increased risk of adverse events when undergoing amyloid lowering treatments. *APOE* genotyping is considered the gold standard for determination of ApoE4 zygosity. Recently, proteotype assays for ApoE4 have become available for high‐throughput immunoassay analyzers. These assays utilize the same specimen type as AD blood biomarkers and can potentially offer improved turnaround times compared to genotyping. In this study, we compare the performance of two automated ApoE4 proteotype research use only (RUO) immunoassays against *APOE* genotyping.

**Method:**

EDTA‐plasma samples (*n* = 65) from individuals undergoing ApoE PCR‐based genotyping for clinical purposes were evaluated. Samples were tested with the Beckman Coulter APOE ε4 RUO assay on the Access 2 and DXI 9000 instruments (Beckman Coulter, Inc), which provides algorithm calculated results for determining ApoE4 zygosity. Manufacturer generated data from 300 genotyped samples was used to calculate estimated cut‐points for ApoE4 zygosity for this study with results <0.55 classified as no E4, ≥0.55 to <4.55 as heterozygous E4, and ≥4.55 as homozygous E4. Samples were also tested with the Lumipulse G ApoE4 and Pan‐ApoE RUO assays on the Fujirebio Lumipulse G1200 analyzer. The ratio of ApoE4/Pan‐ApoE was utilized for determining zygosity with results <5% classified as no E4, ≥5% to <75% as heterozygous E4, and ≥75% as homozygous E4, as listed in the manufacturer IFU.

**Result:**

*APOE* genotype distributions were (n, %): ε3ε4 (30, 46%), ε3ε3 (18, 28%), ε4ε4 (12, 18%), ε2ε3 (3, 5%), ε2ε4 (2, 3%). ApoE4 zygosity was categorized as no ε4 (21, 32%), heterozygous ε4 (32, 49%), or homozygous ε4 (12, 19%). The Beckman Coulter APOE ε4 RUO assay classified 65/65 (100%) patients correctly using manufacturer generated cut‐points on both instruments. The Lumipulse G ApoE4 and Pan‐ApoE RUO assays classified 61/65 (94%) patients correctly using the manufacturer listed cut‐points.

**Conclusion:**

ApoE4 proteotype immunoassays may represent simplified and more accessible testing alternatives to genotyping for the assessment of ApoE4 zygosity in individuals being considered for amyloid lowering treatments.

